# Association between daily eating frequency and mortality in people with diabetes: Findings from NHANES 1999–2014

**DOI:** 10.3389/fnut.2023.937771

**Published:** 2023-01-19

**Authors:** Jing Xie, Zhenwei Wang, Xin Zhang, Junjie Wang, Wei Feng, Yifang Hu, Naifeng Liu, Yun Liu

**Affiliations:** ^1^College of Basic Medicine and Clinical Pharmacy, China Pharmaceutical University, Nanjing, China; ^2^Department of Cardiology, Zhongda Hospital, School of Medicine, Southeast University, Nanjing, China; ^3^Department of Information, The First Affiliated Hospital of Nanjing Medical University, Nanjing, China; ^4^Department of Medical Informatics, School of Biomedical Engineering and Informatics, Nanjing Medical University, Nanjing, China; ^5^Department of Geriatric Endocrinology, The First Affiliated Hospital of Nanjing Medical University, Nanjing, China; ^6^Institute of Medical Informatics and Management, Nanjing Medical University, Nanjing, China

**Keywords:** diabetes, eating frequency, all-cause mortality, CVD-related mortality, National Health and Nutrition Examination Survey

## Abstract

**Background:**

Previous studies have shown that increasing the frequency of eating is beneficial in terms of cardiovascular metabolic risk factors; however, limited evidence is available for the association between daily eating frequency and mortality, especially in people with diabetes. Therefore, we aimed to explore the association between eating frequency and long-term mortality in populations with diabetes.

**Methods:**

We selected 4,924 individuals suffering from diabetes (mean age: 57.77 years; 51.3% men) from the National Health and Nutrition Examination Survey (NHANES) between 1999 and 2014. Daily eating frequency was used as the exposure factor in this study. We extracted the mortality data from the National Death Index records and matched them with the population of NHANES. All participants were followed up from the date of getting enrolled in NHANES to 31 December 2015. Multivariate Cox proportional hazards regression, Kaplan–Meier survival curves, and restricted cubic spline were used to assess the associations between eating frequency and all-cause and cause-specific mortality among people with diabetes.

**Results:**

During 34,950 person–years of follow-up, 1,121 deaths were documented, including 272 cardiovascular disease (CVD)-related deaths and 156 cancer-related deaths. After adjusting for confounding factors, the daily eating frequency was linearly inversely associated with all-cause and CVD-related mortality, and the HR (95% CIs) for per one-time increment of eating frequency was 0.88 (0.80–0.98) and 0.77 (0.63–0.93), respectively. Sensitivity analyses showed that the main results and statistical significance were still stable.

**Conclusion:**

Higher eating frequency was independently related to lower all-cause and CVD-related mortality in people with diabetes, which can be used as a potential strategy for daily-diet management among populations suffering from diabetes.

## 1. Introduction

With the improvement of global economic conditions and the diversification of diet in recent years, more people are suffering from diabetes. The number of people with diabetes was close to 500 million in 2019. It is expected that the number will reach nearly 600 million by 2030, and by 2045, this number will increase by 51%, and also the number of diabetes-related deaths will reach millions each year, which will pose a huge burden on social, financial, and health systems around the world (1–3). Therefore, it is extremely necessary to identify the controllable factors of diabetes as early as possible to prevent the premature death of patients with diabetes.

As we all know, diet plays a very important role in the daily management of patients with diabetes, and the quality and quantity of each diet may lead to great fluctuations in blood glucose, which in turn leads to the progression of diabetes. However, because people with diabetes are more likely to feel more hungry than ordinary people with normal blood glucose, they eat more frequently than normal people to prevent hypoglycemia, while it is unknown whether the increase in eating frequency will benefit the prognosis of patients with diabetes. There was evidence that Chen et al. (4) conducted a long-term follow-up survey of 6,884 participants from the third National Health and Nutrition Examination Survey (NHANES 1988–1992) and found that after adjusting for confounding factors, participants who ate more frequently every day had a 32% lower risk of cardiovascular disease (CVD) death than those who ate less frequently, which was still consistent among female participants. In addition, another prospective cohort study involving 63,999 eligible participants showed that unrestrained eating, a proxy for diet frequency, timing, and caloric intake, was associated with an increased risk of all-cause and cancer-specific mortality, but a decreased risk of cardiovascular disease-specific mortality (5). Although Carew et al. (6) found that the frequency of eating was associated with numerous established risk and preventative factors for coronary artery disease (CAD) at baseline, there was no direct association with the risk of CAD hospitalization or mortality in the 13,328 adult cohorts in Canada. The evidence regarding the effect of eating frequency remains inconclusive. The reasons for this situation may be the different methods of eating frequency assessment and the heterogeneity of the study population, and they failed to perform a subgroup analysis among participants with diabetes, so the association between eating frequency and mortality in people with diabetes remains unknown.

In addition, because diabetics have a faster metabolism and are prone to hunger, they tend to eat more frequently than individuals without diabetes. As we all know, diabetes not only causes a great economic burden to the country and people but also greatly affects the total mortality rate. However, it is unknown whether the eating frequency of patients with diabetes is also related to the effect of diabetes on mortality, so, in this study, we aimed to use the eating frequency of individuals with diabetes as an exposure variable to evaluate its effect on mortality in a nationally representative sample of American adults from NHANES.

## 2. Materials and methods

### 2.1. Study population

For this analysis, we only included people with diabetes and collected the NHANES data set from 1999 to 2014. According to the American Diabetes Association criteria, NHANES defined diabetes through self-reported diagnosis, use of insulin or oral hypoglycemic medication, fasting glucose ≥7.0 mmol/L, or glycated hemoglobin A1c (HbA1c) ≥6.5% (7). We excluded participants with unreliable dietary recall status or unrealistic total daily calorie intake (<800 or >8,000 kcal for men, <600 or >6,000 kcal for women) (8), and we additionally excluded those who were self-reported as pregnant, having cancer at baseline, or were less than 18 years of age. We also excluded participants with ineligibility status for mortality. Finally, we had an analytic sample of 4,924 participants (Supplementary Figure 1). All participants provided written informed consent, and the study protocol was approved by the National Center for Health Statistics of the Center for Disease Control and Prevention Institutional Review Board and in line with the Declaration of Helsinki.

### 2.2. Covariates

Demographic characteristics, health behaviors, dietary habits, medical history, and clinical indicators were considered covariates. Education level was categorized as <9th grade, 9–11th grade, 12th grade, and >12th grade. The family income-to-poverty ratio was classified as 0–1.0, 1.0–3.0, or >3.0. Smoking status was classified as never smoker, former smoker, or current smoker. Alcohol users were defined as those who had at least 12 drinks in the last 12 months. Ideal physical activity was defined as ≥150 min of moderate-intensity activities per week, ≥75 min of vigorous-intensity activities per week, or an equivalent combination of both. activities per week, or an equivalent combination Body mass index (BMI) was calculated as weight in kilograms divided by height in meters squared. Systolic blood pressure (SBP) and diastolic blood pressure (DBP) were measured using a sphygmomanometer after people had rested in a seated position for 5 min. Healthy eating index (HEI), total daily calorie intake, breakfast consumption, and the day of intake were obtained from the 24-h dietary recall interviews, and HEI scores were calculated by R language using the “hei” package. Medical history was self-reported using interviewer-administered questionnaires. Clinical indicators, including fasting glucose, insulin, HbA1c, triglyceride, total cholesterol (TC), low-density lipoprotein cholesterol (LDL-C), and high-density lipoprotein cholesterol (HDL-C) were measured in the NHANES laboratory. Insulin resistance (HOMA2-IR) was calculated with the HOMA calculator (University of Oxford, Oxford, UK) (9), and the estimated glomerular filtration rate (eGFR) was calculated using the Chronic Kidney Disease Epidemiology Collaboration (CKD- EPI) study equation based on serum creatinine (10).

### 2.3. Determination of eating frequency

In this study, eating frequency was defined as the number of eating episodes per day, and an eating episode was defined as food or beverage items (>5 kcal) consumed within 15 min of one another over the 24-h recall (11). The frequency of eating was calculated from the dietary data: Individual Foods files. Detailed information about the time and energy of each occasion reported by each participant is included in the Individual Foods files. From 1999 to 2002, only one dietary recall interview was conducted, but from 2003 to 2014, a second dietary recall interview was added approximately 3–10 days after the first recall. Dietary data from the first recall were included in the present analysis (12).

### 2.4. Ascertainment of mortality

We obtained mortality data for NHANES (1999–2014) from the public-use linked mortality files (LMFs), and the LMFs provide mortality follow-up data from the date of survey participation through 31 December 2015. Mortality outcomes of interest include all-cause, CVD-, and cancer-related mortality. Follow-up time for the present study was defined as the period between the first dietary interview date and the last known date about each participant, living or dead (13).

### 2.5. Statistical analysis

Dietary sampling weights were used in the statistical analysis, and new weights were created for the combined NHANES cycles, as recommended by the National Center for Health Statistics (NCHS). Total eating frequency was categorized as <3, =3, =4, and >4/day (14). Baseline characteristics were presented as mean ± SE or proportions. To calculate the differences between various groups, the weighted Chi-square test was used for categorical variables and the weighted linear regression model was used for continuous variables (15). To examine the associations of eating frequency levels with cardiometabolic biomarkers at baseline, the least squares mean and generalized linear models were used. Multivariate Cox proportional hazard models were constructed to obtain the hazard ratios (HRs) to evaluate the risk of all-cause, CVD-, and cancer-related mortality. In the multivariate models, we adjusted for age, gender, and race/ethnicity in model 1. In model 2, we further adjusted for BMI, education level, family income–poverty ratio, alcohol user, smoking status, ideal physical activity, healthy eating index (HEI) scores, daily calorie intake, breakfast skipping, and diet record days. In model 3, we further adjusted for duration of diabetes, diabetes medication use (16), self-reported hypertension, hypercholesterolemia, and CVDs, and self-reported hypertension and hypercholesterolemia medication use. In model 4, we further adjusted for HbA1c, HOMA2-IR, SBP, DBP, TC, triglyceride, HDL-C, LDL-C, and eGFR. Multiple imputations were used for missing covariates. The linear trend was tested by assigning a median value to each category as a continuous variable.

Restricted cubic spline regression with three knots (5th, 50th, and 75th) was used to check if there existed a non-linear relationship between the frequency of eating and mortality. Stratified analyses were also conducted by age (≤65 or >65 years), gender (male or female), race/ethnicity (White or non-White), alcohol use (Yes or No), ideal physical activity (Yes or No), smoking status (never, ever, current smoker), BMI (<30.0 or ≥30.0 kg/m^2^), and diabetes duration (≤10 or >10 years). The *p*-values for the product terms between frequency of eating and stratification variables were used to estimate the significance of interactions. Sensitivity analyses were performed to test the robustness of our findings. First, to verify the stability of the results, we excluded participants with unrealistic total daily calorie intake (<500 or >3,500 kcal/day for women, and <800 or >4,200 kcal/day for men) based on an existing reference (17). Second, to reduce the potential reverse causation bias, we excluded those who died within the first 1 or 2 years of follow-up. All statistical analyses were performed in R software (4.1.0).

## 3. Results

### 3.1. Baseline characteristics by quartile of eating frequency

Among the 4,924 participants with diabetes (mean age: 57.77 years; 51.3% male), the daily eating frequency ranged from 1 to 8. The baseline characteristics of the different eating frequency subgroups are shown in Table 1. Compared with participants who ate less frequently (eating frequency <3 times), participants who ate more frequently (eating frequency > 4 times) were more non-Hispanic-White, more alcohol users, more ever smokers, more people with higher education levels and family income–poverty ratio, had more ideal physical activity, had higher daily calorie intake and healthy eating index (HEI) score, more people who ate breakfast and had diet record in weekdays, and had more hypercholesterolemia medication use (all *P* < 0.05). Importantly, participants with more eating frequency had lower all-cause and CVD-related mortality (all *P* < 0.01). Table 2 shows the least squares means of cardiac metabolic risk factors grouped based on eating frequency. Participants who ate more frequently had lower levels of HOMA2-IR than those who ate less (*P* = 0.014), and there was no significant association between eating frequency and glucose, insulin, HbA1c, TC, HDL-C, LDL-C, triglycerides, and eGFR (all *P* > 0.05).

**TABLE 1:** Baseline characteristics of participants with diabetes classified according to eating frequency in National Health and Nutrition Examination Survey (NHANES) 1999–2014.

	Total	Eating frequency < 3	Eating frequency = 3	Eating frequency = 4	Eating frequency > 4	*P*-value
Age (mean ± SE) (years)	57.77 ± 0.33	54.84 ± 1.01	57.78 ± 0.56	58.38 ± 0.41	57.09 ± 0.78	0.004
Gender						0.005
Male	2,526 (51.3)	174 (58.0)	736 (50.7)	1,129 (48.5)	487 (57.7)	
Female	2,398 (48.7)	126 (42.0)	717 (49.3)	1,199 (51.5)	356 (42.3)	
Race/Ethnicity						<0.001
Mexican American	491 (10.0)	32 (10.8)	162 (11.1)	206 (8.8)	92 (10.9)	
Other Hispanic	316 (6.4)	20 (6.8)	102 (7.1)	157 (6.7)	36 (4.3)	
Non-Hispanic white	2,883 (58.6)	132 (44.2)	784 (54.0)	1,418 (60.9)	548 (65.0)	
Non-Hispanic black	839 (17.0)	97 (32.4)	309 (21.3)	350 (15.0)	83 (9.8)	
Other race	394 (8.0)	17 (5.8)	96 (6.6)	197 (8.5)	84 (9.9)	
BMI (mean ± SE) (kg/m^2^)	33.02 ± 0.18	34.02 ± 0.55	33.08 ± 0.32	32.89 ± 0.24	32.92 ± 0.47	0.248
SBP (mean ± SE) (mmHg)	131.25 ± 0.48	133.37 ± 1.59	130.98 ± 0.72	130.91 ± 0.63	131.89 ± 1.50	0.407
DBP (mean ± SE) (mmHg)	69.91 ± 0.41	72.82 ± 0.96	70.03 ± 0.70	68.94 ± 0.52	71.35 ± 0.85	<0.001
Alcohol user						0.030
Yes	3,245 (65.9)	192 (63.9)	933 (64.2)	1,513 (65.0)	607 (72.0)	
No	1,679 (34.1)	108 (36.1)	520 (35.8)	815 (35.0)	236 (28.0)	
Smoking status						0.032
Never smoker	2,411 (49.0)	133 (44.3)	690 (47.5)	1,207 (51.9)	380 (45.0)	
Ever smoker	1,678 (34.1)	94 (31.2)	516 (35.5)	773 (33.2)	295 (35.0)	
Current smoker	836 (17.0)	73 (24.5)	246 (16.9)	348 (14.9)	169 (20.0)	
Education levels						<0.001
<9th grade	616 (12.5)	44 (14.6)	223 (15.3)	255 (11.0)	94 (11.1)	
9–11th grade	850 (17.3)	76 (25.4)	279 (19.2)	381 (16.4)	114 (13.5)	
12th grade	1,273 (25.9)	89 (29.8)	364 (25.1)	596 (25.6)	224 (26.5)	
>12th grade	2,185 (44.4)	91 (30.2)	587 (40.4)	1,095 (47.0)	412 (48.9)	
Family income-poverty ratio						<0.001
≤1.0	961 (19.5)	101 (33.5)	352 (24.2)	370 (15.9)	138 (16.3)	
1.0–3.0	2,079 (42.2)	116 (38.7)	636 (43.8)	973 (41.8)	354 (41.9)	
>3.0	1,884 (38.3)	83 (27.8)	465 (32.0)	984 (42.3)	352 (41.8)	
Ideal physical activity						0.011
Yes	1,905 (38.7)	79 (26.3)	543 (37.4)	946 (40.7)	336 (39.9)	
No	3,019 (61.3)	221 (73.7)	910 (62.6)	1,381 (59.3)	507 (60.1)	
HEI score, (mean ± SE)	55.80 ± 0.31	50.47 ± 1.04	53.96 ± 0.47	57.39 ± 0.38	56.48 ± 0.73	<0.001
Daily calorie intake (mean ± SE), calories	1,976.15 ± 17.93	1,557.59 ± 56.46	1,807.98 ± 30.57	2,015.04 ± 24.98	2,307.35 ± 47.95	<0.001
Breakfast skipping						<0.001
Yes	964 (19.6)	182 (60.7)	417 (28.7)	276 (11.9)	89 (10.5)	
No	3,960 (80.4)	118 (39.3)	1,036 (71.3)	2,052 (88.1)	755 (89.5)	
Diet record days						0.012
Weekdays	4,292 (87.2)	249 (82.9)	1,240 (85.4)	2,038 (87.5)	765 (90.8)	
Non-weekdays	632 (12.8)	51 (17.1)	213 (14.6)	290 (12.5)	78 (9.2)	
Duration of diabetes						0.815
≤3 years	1,568 (31.8)	102 (34.1)	458 (31.5)	732 (31.4)	277 (32.8)	
3–10 years	1,662 (33.7)	90 (29.9)	511 (35.2)	795 (34.1)	267 (31.6)	
>10 years	1,694 (34.4)	108 (36.1)	484 (33.3)	802 (34.4)	300 (35.6)	
Hypertension						0.987
Yes	3,047 (61.9)	189 (62.9)	899 (61.9)	1,442 (62.0)	517 (61.3)	
No	1,877 (38.1)	111 (37.1)	554 (38.1)	886 (38.0)	326 (38.7)	
Hypercholesterolemia						0.118
Yes	2,772 (56.3)	152 (50.8)	800 (55.0)	1,308 (56.2)	512 (60.7)	
No	2,152 (43.7)	147 (49.2)	653 (45.0)	1,020 (43.8)	331 (39.3)	
CVD						0.884
Yes	1,171 (23.8)	69 (22.9)	358 (24.6)	553 (23.8)	192 (22.7)	
No	3,753 (76.2)	231 (77.1)	1,095 (75.4)	1,775 (76.2)	652 (77.3)	
Diabetes medication use						0.075
No insulin or pills	1,750 (35.5)	141 (47.2)	553 (38.1)	773 (33.2)	282 (33.5)	
Only diabetes pills	2,150 (43.7)	112 (37.3)	607 (41.8)	1,058 (45.4)	373 (44.3)	
Only insulin	549 (11.1)	22 (7.2)	167 (11.5)	268 (11.5)	92 (10.9)	
Pills and insulin	475 (9.6)	25 (8.3)	126 (8.6)	229 (9.8)	96 (11.4)	
Hypertension medication use						0.827
Yes	2,645 (53.7)	152 (50.6)	779 (53.6)	1,264 (54.3)	450 (53.4)	
No	2,279 (46.3)	148 (49.4)	674 (46.4)	1,064 (45.7)	393 (46.6)	
Hypercholesterolemia medication use						0.003
Yes	2,009 (40.8)	85 (28.4)	573 (39.4)	974 (41.9)	377 (44.7)	
No	2,915 (59.2)	215 (71.6)	880 (60.6)	1,354 (58.1)	467 (55.3)	
**Outcomes**						
All-cause mortality						<0.001
Yes	988 (20.1)	70 (23.2)	341 (23.5)	459 (19.7)	118 (14.0)	
No	3,936 (79.9)	230 (76.8)	1,112 (76.5)	1,869 (80.3)	725 (86.0)	
CVD-related mortality						<0.001
Yes	242 (4.9)	21 (7.0)	101 (7.0)	91 (3.9)	28 (3.3)	
No	4,682 (95.1)	279 (93.0)	1,352 (93.0)	2,236 (96.1)	815 (96.7)	
Cancer-related mortality						0.170
Yes	134 (2.7)	6 (2.1)	42 (2.9)	73 (3.1)	13 (1.6)	
No	4,790 (97.3)	293 (97.9)	1,411 (97.1)	2,255 (96.9)	830 (98.4)	

Data are numbers (percentages) unless otherwise indicated. All estimates accounted for complex survey designs.

**TABLE 2:** Least squares mean of cardiometabolic markers according to eating frequency among diabetes.

	Eating frequency < 3	Eating frequency = 3	Eating frequency = 4	Eating frequency > 4	*P* trend
Glucose (mmol/L)	9.43 ± 0.36	9.13 ± 0.22	9.01 ± 0.22	8.71 ± 0.27	0.101
Insulin (pmol/L)	162.89 ± 20.56	138.99 ± 8.52	144.24 ± 9.68	136.94 ± 26.95	0.686
HOMA2-IR	3.28 ± 0.36	2.91 ± 0.24	2.93 ± 0.17	2.37 ± 0.23	0.014
HbA1c (%)	7.38 ± 0.14	7.55 ± 0.08	7.50 ± 0.07	7.35 ± 0.09	0.125
Total cholesterol (mmol/L)	4.75 ± 0.13	4.80 ± 0.07	4.72 ± 0.06	4.71 ± 0.08	0.350
HDL-C (mmol/L)	1.29 ± 0.05	1.21 ± 0.02	1.18 ± 0.02	1.20 ± 0.03	0.149
LDL-C (mmol/L)	2.69 ± 0.12	2.71 ± 0.07	2.67 ± 0.07	2.72 ± 0.1	0.947
Triglyceride (mmol/L)	2.09 ± 0.19	2.32 ± 0.13	2.38 ± 0.11	2.51 ± 0.22	0.185
eGFR, ml/min 1.73m2	81.65 ± 1.75	82.24 ± 0.84	83.68 ± 0.82	83.52 ± 1.17	0.152

The least square (mean ± SE), and *P* trend were estimated with adjustment of age, sex, race/ethnicity, BMI, education level, family income-poverty ratio, alcohol user, smoking status, ideal physical activity, healthy eating index (HEI) score, daily calorie intake, breakfast skipping, diet record days, duration of diabetes, diabetes medication use, self-reported hypertension, hypercholesterolemia, and CVD, and self-reported hypertension, hypercholesterolemia medication use.

### 3.2. Association between eating frequency and mortality

**FIGURE 1:**
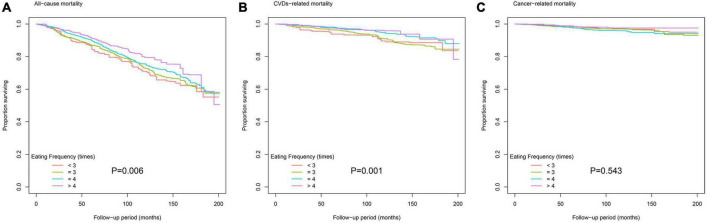
Kaplan–Meier survival curve for **(A)** all-cause mortality, **(B)** cardiovascular disease (CVD)-related mortality, and **(C)** cancer-related mortality according to eating frequency.

**FIGURE 2:**
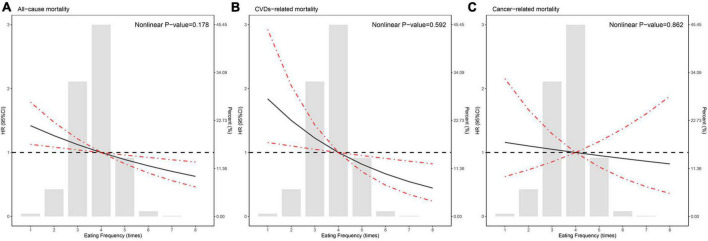
Hazard ratios for **(A)** all-cause mortality, **(B)** cardiovascular disease (CVD)-related mortality, and **(C)** cancer-related mortality according to eating frequency and the histogram of the probability distribution is presented in the background. Hazard ratios were calculated by Cox models after adjusting for age, sex, race/ethnicity, Body mass index (BMI), education level, family income-poverty ratio, alcohol consumption, smoking status, ideal physical activity, healthy eating index (HEI) score, daily calorie intake, breakfast skipping, diet record days, duration of diabetes, diabetes medication use, self-reported hypertension, hypercholesterolemia, and CVD, self-reported hypertension, hypercholesterolemia medication use, HbA1c, HOMA2_IR, systolic blood pressure, diastolic blood pressure, total cholesterol, triglyceride, high-density lipoprotein, low-density lipoprotein, and estimated glomerular filtration rate (eGFR).

During 34,950 person-years of follow-up, 1,121 deaths were documented, including 272 CVD-related deaths and 156 cancer-related deaths. Table 3 shows the results of multivariate Cox proportional hazard regression analyses of the association between eating frequency and mortality. After adjusting for confounding factors including age, sex, race/ethnicity, BMI, education level, family income–poverty ratio, alcohol consumption, smoking status, ideal physical activity, healthy eating index (HEI) score, daily calorie intake, breakfast skipping, diet record days, duration of diabetes, diabetes medication use, self-reported hypertension, hypercholesterolemia, CVDs, self-reported hypertension, hypercholesterolemia medication use, HbA1c, HOMA2-IR, SBP, DBP, TC, triglyceride, HDL-C, LDL-C, and eGFR, regardless of whether the eating frequency was used as a continuous variable or a classified variable, it was significantly associated with all-cause and CVD-related mortality. When eating frequency was used as a continuous variable, the HR was evaluated per one time increment of eating frequency, and HRs (95% CIs) for all-cause and CVD-related mortality were, respectively, 0.88 (0.80–0.98) and 0.77 (0.63–0.93); when eating frequency was used as a classified variable, the HR was evaluated based on a subgroup (eating frequency <3) as a reference, and HRs (95% CIs) of subgroup (eating frequency > 4) for all-cause and CVD-related mortality were, respectively, 0.67 (0.45–1.01) and 0.53 (0.26–1.08); the P trend values were, respectively, 0.013 and 0.005. Additionally, as shown in Figure 1, the Kaplan–Meier survival curve stratified according to the eating frequency showed that the cumulative incidence of all-cause and CVD-related death decreased with the increase in eating frequency (log-rank test, *P* < 0.05). Figure 2 shows the restricted cubic spline results of the association between eating frequency and mortality. We found that there was a linear association between eating frequency and the risk of all-cause and CVD-related mortality (all non-linear *P* > 0.05).

**TABLE 3:** HR (95% CIs) for all-cause and cause-specific mortality according to eating frequency among diabetes.

	Eating frequency (per 1 time increment)	Eating frequency < 3	Eating frequency = 3	Eating frequency = 4	Eating frequency > 4	*P* trend
**All-cause mortality**						
Model 1*	0.85 (0.77, 0.93)	1.00	0.81 (0.55, 1.20)	0.67 (0.47, 0.95)	0.60 (0.41, 0.89)	<0.001
Model 2^†^	0.89 (0.80, 0.99)	1.00	0.94 (0.59, 1.49)	0.81 (0.51, 1.28)	0.71 (0.44, 1.15)	0.028
Model 3^‡^	0.89 (0.80, 0.99)	1.00	0.98 (0.62, 1.56)	0.84 (0.53, 1.34)	0.73 (0.45, 1.18)	0.032
Model 4^§^	0.88 (0.80, 0.98)	1.00	0.89 (0.61, 1.29)	0.77 (0.54, 1.11)	0.67 (0.45, 1.01)	0.013
**CVD-related mortality**						
Model 1*	0.72 (0.61, 0.86)	1.00	0.84 (0.45, 1.54)	0.46 (0.26, 0.83)	0.49 (0.26, 0.94)	<0.001
Model 2^†^	0.80 (0.66, 0.96)	1.00	1.01 (0.53, 1.93)	0.61 (0.32, 1.16)	0.67 (0.34, 1.35)	0.015
Model 3^‡^	0.78 (0.64, 0.95)	1.00	1.02 (0.52, 1.98)	0.61 (0.31, 1.19)	0.62 (0.30, 1.29)	0.009
Model 4^§^	0.77 (0.63, 0.93)	1.00	0.89 (0.49, 1.60)	0.54 (0.30, 0.97)	0.53 (0.26, 1.08)	0.005
**Cancer-related mortality**						
Model 1*	0.92 (0.75, 1.13)	1.00	1.13 (0.46, 2.83)	1.21 (0.49, 3.01)	0.71 (0.27, 1.87)	0.416
Model 2^†^	0.90 (0.72, 1.13)	1.00	1.08 (0.42, 2.76)	1.13 (0.45, 2.89)	0.67 (0.24, 1.85)	0.360
Model 3^‡^	0.92 (0.74, 1.15)	1.00	1.24 (0.49, 3.11)	1.32 (0.52, 3.36)	0.77 (0.27, 2.16)	0.485
Model 4^§^	0.91 (0.73, 1.14)	1.00	1.17 (0.46, 2.98)	1.25 (0.48, 3.22)	0.73 (0.26, 2.05)	0.430

*Model 1: Adjusted for age, sex, and race/ethnicity; ^†^Model 2: Further adjusted (from Model 1) for BMI, education level, family income-poverty ratio, alcohol user, smoking status, ideal physical activity, healthy eating index (HEI) score, daily calorie intake, breakfast skipping, and diet record days; ^‡^Model 3: Further adjusted (from Model 2) for duration of diabetes, diabetes medication use, self-reported hypertension, hypercholesterolemia, and CVD, and self-reported hypertension, hypercholesterolemia medication use; ^§^ Model 4: Further adjusted (from Model 3) for HbA1c, HOMA2_IR, systolic blood pressure, diastolic blood pressure, total cholesterol, triglyceride, high-density lipoprotein, low-density lipoprotein, and estimated glomerular filtration rate (eGFR).

Sensitivity analyses showed that the main results and statistical significance were still consistent with Table 3 where participants with unreliable dietary recall status or unrealistic total daily calorie intake (<500 or >3,500 kcal/day for women, and <800 or >4,200 kcal/day for men), or participants who died during the 1-year follow-up period were excluded (Supplementary Tables 1, 2). However, when we excluded individuals who died within 2 years of follow-up, the association of eating frequency with all-cause mortality and CVD-related mortality in participants with diabetes was weakened or even no longer significant (Supplementary Table 3).

### 3.3. Subgroup analyses

Supplementary Table 4 showed stratified analyses of the association between eating frequency and all-cause mortality among participants with diabetes. The results showed that there was a negative association between eating frequency and all-cause mortality in the subgroups of age ≤65 years, male, non-White, alcohol drinking, having ideal physical activity, never smoking, BMI > 30kg/m^2^, and diabetes duration of ≤10 years. Stratified analyses of the association between eating frequency and CVD-related mortality in participants with diabetes are shown in Supplementary Table 5, and we found that eating frequency was negatively associated with CVD-related mortality in participants with age >65 years, male, non-White, alcohol drinking, with and without ideal physical activity, never and current smoking, BMI ≤ 30kg/m^2^, and BMI > 30kg/m^2^. No significant interaction was found between eating frequency and these stratification variables for both all-cause mortality and CVD mortality.

## 4. Discussion

Our study filled the knowledge gap of the association between eating frequency and mortality in people with diabetes. In this study, we found eating frequency was linearly inversely associated with all-cause and CVD-related mortality in people with diabetes, and this association was stable in many subgroups. These findings added some potentially effective clues to the daily diet management of patients with diabetes.

As early as the 1960s, Fábry et al. (18) found that the increase in eating frequency could not only change the overall energy metabolism and increase the production of glycogen and fat in rats but also improve the overweight, blood lipid, and glucose tolerance of normal people. However, the eating frequency they defined only came from the records of meals, not including the intake times of snacks and other non-meals. Subsequently, Stote et al. (19) showed in an 8-week randomized controlled trial that at the end of the experiment, when the total daily calorie intake of the two groups was the same, compared with the subjects who ate 1 meal/day, the SBP, DBP, TC, and LDL-C of the subjects who ate 3 meals/days were lower, while the urea nitrogen was higher, which indicated that the change of eating frequency could lead to the adaptive changes of some cardiovascular metabolic factors without affecting the daily energy needs of the body. In addition, several observational studies have proved that higher eating frequency can improve blood lipids, blood pressure, BMI, and waist circumference (14, 20–25). However, a recent review, after summarizing the results of nine previous clinical trials drew an opposite conclusion, that is, without changing the daily total calorie intake, increasing the frequency of eating might not help improve the traditional metabolic risk factors of CVDs or help in losing weight (26). Besides, another epidemiological study showed that the increase in eating frequency among children aged 9–10 was related to poor diet quality, bad behavior, and obesity (27). However, there was also no consistent conclusion in clinical diseases. For instance, there were pieces of evidence that higher eating frequency was associated with lower risks of metabolic syndrome and hypertension (28–30), whereas another study demonstrated that higher frequency of eating was related to the progression rate of blood pressure and new-onset hypertension among adults free from CVDs and diabetes (31). At present, the studies on the associations between eating frequency and dietary quality and cardiovascular metabolic markers are still widely concerned, while the report on the association between eating frequency and mortality is not only rare but also has no unified conclusion. For example, in a cohort study of 13,328 participants from Canada (2004–2013), Carew et al. (6) found that there was no direct association between eating frequency and the risk of CAD mortality. Nevertheless, another cohort survey involving 6,884 participants from NHANES (1988–1992) showed that more eating frequency was closely associated with lower CVD-related mortality in the fully adjusted model (4). Moreover, not only eating frequency is associated with mortality but a previous study has also shown that inappropriate eating environments may also be associated with other diet-related health problems, such as increased intake of hyper-processed foods (32). Although our study found no differences in glucose, insulin, serum lipid, and renal function among different groups of eating frequency, we got another exciting result, that is, there was a significant negative association between eating frequency and insulin resistance among patients with diabetes.

Although this study has achieved the expected results, the mechanism was still unclear. After investigating the related literature, we found that there might be several mechanisms to mediate the association between eating frequency and mortality. For example, several similar studies have found that increasing the frequency of eating can improve the fat production and insulin resistance of mice and the cardiovascular metabolic risk factors of subjects (14, 18, 20, 21, 24, 33, 34), and the reduction of these risk factors can benefit the long-term prognosis. Additionally, Zhang et al. (35) found in a large cohort study that an uncontrolled diet is associated with an increase in all-cause mortality and cancer-related mortality (especially gastrointestinal cancer), but is associated with a decrease in CVD-related mortality, while insulin resistance is closely associated with mortality, which suggests that the insulin resistance pathway may be potentially important in the relationship between dietary behavior and major health outcomes (5). In addition, some studies have shown that the increase in the frequency of eating is related to the improvement in the quality of diet (22, 25, 36–38), and the daily diet quality is closely related to human health, which may indirectly improve the long-term death risk. Finally, metabolic syndrome, obesity, and hypertension are recognized independent risk factors of CVDs and deaths, while previous studies have shown that higher eating frequency is associated with a lower prevalence of metabolic syndrome, obesity, and hypertension (28, 29), which explained why increasing the frequency of eating can reduce all-cause and CVD-related mortality. However, most of the above possible mechanisms come from randomized controlled trials and epidemiological investigations, while related cell and animal tests are still rare, so more basic studies are needed to determine the potential mechanisms.

Despite the exciting findings of this study, there were still several limitations. For example, as an observational study, we were unable to determine the causal link between eating frequency and mortality. Additionally, there was currently no accepted standard for the definition of eating frequency, and we only referred to previous studies to define eating frequency, so the promotion of the results was limited to people with a similar eating frequency. Furthermore, in this study, due to the limited NHANES data, the assessment of eating frequency and other covariates is a one-off, and there are no repeated measurements, so we were unable to assess the impact of dynamic changes in eating frequency on mortality. In addition, although we took into account the nature of the day in which participants ate, such as weekdays or weekends, we did not assess the main places where eating frequency occurred, and eating frequency too much outside might increase mortality. Also, we failed to assess the effect of snack frequency on eating frequency in our analysis, which was also a restriction of our study. Moreover, we failed to explore the association between eating frequency and mortality in other populations, such as those without diabetes or those prone to hypoglycemia. Although according to the current data, we are temporarily unable to determine why dietary frequency can help diabetics prevent premature death, and the mechanism is unknown, our findings in this study also provided some reference and theoretical basis for the management of diabetes patients. As we all know, the management of patients with diabetes is complicated. The diet of patients with diabetes is not only related to the frequency of diet but also the quantity and type of food. When we analyze the impact of dietary factors on mortality in patients with diabetes, we should fully consider all dietary factors, including food type, composition, and frequency of eating, but our data do not have such detailed dietary data. Therefore, we can only analyze the relationship between eating frequency and mortality in patients with diabetes, which is one of the limitations of our study. Finally, we might not be able to control some non-man-made confounding factors, such as genetic susceptibility.

## 5. Conclusion

We found that higher eating frequency was related to lower all-cause and CVD-related mortality in people with diabetes, and the association between eating frequency and mortality was independent of overall diet quality, total calorie intake, and the timing of eating, which can be used as a potential strategy for the daily diet management among populations suffering from diabetes, as well as providing valuable insights for formulating preventive measures against premature death.

## Data availability statement

The original contributions presented in this study are included in the article/Supplementary material, further inquiries can be directed to the corresponding authors.

## Ethics statement

The National Center for Health Statistics of the Center for Disease Control and Prevention Institutional Review Board. The patients/participants provided their written informed consent to participate in this study.

## Author contributions

JX and ZW conducted the analyses and wrote the first draft of the manuscript. XZ, JW, WF, and YH collected and assembled the data. NL and YL conceived the study design. All authors contributed to the interpretation of the results and the critical revision of the manuscript for important intellectual content and read and approved the final version of the manuscript.
