# Supplementary material for: Association between daily eating frequency and mortality in people with diabetes: Findings from NHANES 1999–2014
Source: Front Nutr. 2023 Jan 19;10:937771. doi: 10.3389/fnut.2023.937771 (PMC9894317; doi:10.3389/fnut.2023.937771)
Supplement: Supplementary file 1 [file Data_Sheet_1.docx]

# Supplementary Table 1. Hazard ratios (95% CIs) of all-cause and cause-specific mortality according to eating frequency among diabetes after excluding participants with unrealistic total daily calorie intake (<500 or >3500 kcal/d for females, and <800 or >4200 kcal/d for males) (n = 4811)

|  | Eating frequency  (per 1 time increment) | Eating frequency < 3 | Eating frequency = 3 | Eating frequency = 4 | Eating frequency > 4 | P trend |
| --- | --- | --- | --- | --- | --- | --- |
| All-cause mortality | | | | | | |
| Model 1* | 0.84 (0.77,0.92) | 1.00 | 0.82 (0.56,1.21) | 0.67 (0.47,0.96) | 0.59 (0.40,0.86) | <0.001 |
| Model 2† | 0.88 (0.80,0.98) | 1.00 | 0.95 (0.60,1.50) | 0.82 (0.52,1.29) | 0.70 (0.44,1.11) | 0.018 |
| Model 3‡ | 0.88 (0.80,0.98) | 1.00 | 0.99 (0.63,1.57) | 0.86 (0.54,1.36) | 0.71 (0.45,1.14) | 0.020 |
| Model 4§ | 0.88 (0.80,0.97) | 1.00 | 0.90 (0.62,1.32) | 0.79 (0.55,1.14) | 0.66 (0.44,0.99) | 0.008 |
| CVDs-related mortality | | | | | | |
| Model 1* | 0.73 (0.61,0.86) | 1.00 | 0.82 (0.45,1.51) | 0.46 (0.26,0.83) | 0.50 (0.26,0.96) | <0.001 |
| Model 2† | 0.80 (0.66,0.97) | 1.00 | 0.99 (0.52,1.88) | 0.61 (0.32,1.16) | 0.67 (0.33,1.36) | 0.020 |
| Model 3‡ | 0.78 (0.64,0.95) | 1.00 | 1.00 (0.51,1.93) | 0.61 (0.31,1.19) | 0.61 (0.29,1.29) | 0.012 |
| Model 4§ | 0.77 (0.63,0.94) | 1.00 | 0.88 (0.49,1.57) | 0.55 (0.30,0.98) | 0.53 (0.26,1.09) | 0.006 |
| Cancer-related mortality | | | | | | |
| Model 1* | 0.93 (0.75,1.14) | 1.00 | 1.14 (0.46,2.85) | 1.22 (0.49,3.04) | 0.74 (0.28,1.96) | 0.479 |
| Model 2† | 0.90 (0.71,1.13) | 1.00 | 1.07 (0.41,2.76) | 1.12 (0.44,2.88) | 0.67 (0.24,1.87) | 0.357 |
| Model 3‡ | 0.92 (0.74,1.15) | 1.00 | 1.23 (0.48,3.11) | 1.30 (0.51,3.34) | 0.78 (0.27,2.20) | 0.494 |
| Model 4§ | 0.91 (0.73,1.14) | 1.00 | 1.16 (0.45,2.97) | 1.23 (0.47,3.18) | 0.73 (0.26,2.06) | 0.429 |

Note:

* Model 1: adjusted for age, sex and race/ethnicity;

† Model 2: further adjusted (from Model 1) for BMI, education level, family income-poverty ratio, alcohol user, smoking status, ideal physical activity, healthy eating index (HEI) score, daily calorie intake, breakfast skipping, and diet record days;

‡ Model 3: further adjusted (from Model 2) for duration of diabetes, diabetes medication use, self-reported hypertension, hypercholesterolemia, and CVD, and self-reported hypertension, hypercholesterolemia medication use;

§ Model 4: further adjusted (from Model 3) for HbA1c, HOMA2_IR, systolic blood pressure, diastolic blood pressure, total cholesterol, triglyceride, high density lipoprotein, low density lipoprotein, and eGFR.

# Supplementary Table 2. Hazard ratios (95% CIs) of all-cause and cause-specific mortality according to eating frequency among diabetes after excluding participants who died within first one year of follow-up (n = 4849)

|  | Eating frequency  (per 1 time increment) | Eating frequency < 3 | Eating frequency = 3 | Eating frequency = 4 | Eating frequency > 4 | P trend |
| --- | --- | --- | --- | --- | --- | --- |
| All-cause mortality | | | | | | |
| Model 1^*^ | 0.85 (0.77,0.93) | 1.00 | 0.85 (0.56,1.27) | 0.69 (0.47,1.02) | 0.59 (0.39,0.89) | <0.001 |
| Model 2^†^ | 0.88 (0.79,0.98) | 1.00 | 0.95 (0.59,1.55) | 0.81 (0.50,1.32) | 0.68 (0.41,1.12) | 0.014 |
| Model 3^‡^ | 0.88 (0.79,0.98) | 1.00 | 1.00 (0.62,1.62) | 0.85 (0.52,1.39) | 0.70 (0.42,1.15) | 0.019 |
| Model 4^§^ | 0.87 (0.78,0.96) | 1.00 | 0.87 (0.60,1.25) | 0.75 (0.52,1.07) | 0.61 (0.41,0.92) | 0.004 |
| CVDs-related mortality | | | | | | |
| Model 1^*^ | 0.73 (0.63,0.85) | 1.00 | 0.94 (0.50,1.76) | 0.49 (0.27,0.90) | 0.53 (0.28,0.99) | <0.001 |
| Model 2^†^ | 0.80 (0.67,0.96) | 1.00 | 1.13 (0.57,2.21) | 0.63 (0.32,1.24) | 0.70 (0.35,1.42) | 0.010 |
| Model 3^‡^ | 0.78 (0.64,0.95) | 1.00 | 1.13 (0.56,2.30) | 0.63 (0.31,1.29) | 0.64 (0.30,1.38) | 0.007 |
| Model 4^§^ | 0.77 (0.63,0.93) | 1.00 | 0.98 (0.55,1.75) | 0.55 (0.31,1.00) | 0.54 (0.27,1.11) | 0.003 |
| Cancer-related mortality | | | | | | |
| Model 1^*^ | 0.88 (0.71,1.11) | 1.00 | 1.10 (0.44,2.77) | 1.16 (0.46,2.91) | 0.58 (0.20,1.66) | 0.271 |
| Model 2^†^ | 0.88 (0.69,1.12) | 1.00 | 1.11 (0.43,2.89) | 1.16 (0.45,3.04) | 0.59 (0.20,1.79) | 0.299 |
| Model 3^‡^ | 0.90 (0.70,1.14) | 1.00 | 1.26 (0.50,3.21) | 1.34 (0.52,3.47) | 0.66 (0.22,2.03) | 0.371 |
| Model 4^§^ | 0.89 (0.70,1.13) | 1.00 | 1.20 (0.46,3.09) | 1.27 (0.48,3.31) | 0.63 (0.21,1.94) | 0.329 |

Note:

* Model 1: adjusted for age, sex and race/ethnicity;

† Model 2: further adjusted (from Model 1) for BMI, education level, family income-poverty ratio, alcohol user, smoking status, ideal physical activity, healthy eating index (HEI) score, daily calorie intake, breakfast skipping, and diet record days;

‡ Model 3: further adjusted (from Model 2) for duration of diabetes, diabetes medication use, self-reported hypertension, hypercholesterolemia, and CVD, and self-reported hypertension, hypercholesterolemia medication use;

§ Model 4: further adjusted (from Model 3) for HbA1c, HOMA2_IR, systolic blood pressure, diastolic blood pressure, total cholesterol,triglyceride, high density lipoprotein, low density lipoprotein, and eGFR.

# Supplementary Table 3. Hazard ratios (95% CIs) of all-cause and cause-specific mortality according to eating frequency among diabetes after excluding participants who died within first two years of follow-up (n = 4421)

|  | Eating frequency  (per 1 time increment) | Eating frequency < 3 | Eating frequency = 3 | Eating frequency = 4 | Eating frequency > 4 | P trend |
| --- | --- | --- | --- | --- | --- | --- |
| All-cause mortality | | | | | | |
| Model 1^*^ | 0.88 (0.79,0.98) | 1.00 | 0.77 (0.51,1.17) | 0.69 (0.46,1.05) | 0.61 (0.39,0.96) | 0.017 |
| Model 2^†^ | 0.93 (0.82,1.05) | 1.00 | 0.89 (0.55,1.43) | 0.84 (0.51,1.39) | 0.73 (0.43,1.24) | 0.181 |
| Model 3^‡^ | 0.93 (0.82,1.05) | 1.00 | 0.92 (0.56,1.50) | 0.87 (0.52,1.46) | 0.74 (0.43,1.28) | 0.198 |
| Model 4^§^ | 0.91 (0.81,1.02) | 1.00 | 0.76 (0.53,1.08) | 0.72 (0.50,1.03) | 0.62 (0.40,0.97) | 0.069 |
| CVDs-related mortality | | | | | | |
| Model 1^*^ | 0.78 (0.66,0.92) | 1.00 | 0.98 (0.53,1.81) | 0.54 (0.29,1.00) | 0.66 (0.33,1.29) | 0.003 |
| Model 2^†^ | 0.85 (0.70,1.03) | 1.00 | 1.19 (0.62,2.28) | 0.70 (0.35,1.37) | 0.87 (0.41,1.85) | 0.071 |
| Model 3^‡^ | 0.83 (0.68,1.02) | 1.00 | 1.19 (0.58,2.43) | 0.69 (0.33,1.44) | 0.80 (0.35,1.84) | 0.054 |
| Model 4^§^ | 0.82 (0.66,1.01) | 1.00 | 1.01 (0.55,1.86) | 0.59 (0.32,1.11) | 0.68 (0.31,1.48) | 0.028 |
| Cancer-related mortality | | | | | | |
| Model 1^*^ | 0.92 (0.73,1.18) | 1.00 | 0.89 (0.32,2.42) | 1.07 (0.41,2.83) | 0.56 (0.18,1.73) | 0.516 |
| Model 2^†^ | 0.94 (0.72,1.22) | 1.00 | 0.92 (0.33,2.61) | 1.14 (0.41,3.17) | 0.61 (0.19,1.99) | 0.642 |
| Model 3^‡^ | 0.96 (0.74,1.24) | 1.00 | 1.04 (0.37,2.89) | 1.29 (0.47,3.53) | 0.68 (0.21,2.23) | 0.737 |
| Model 4^§^ | 0.93 (0.72,1.21) | 1.00 | 0.91 (0.33,2.52) | 1.13 (0.42,3.03) | 0.60 (0.18,1.95) | 0.599 |

Note:

* Model 1: adjusted for age, sex and race/ethnicity;

† Model 2: further adjusted (from Model 1) for BMI, education level, family income-poverty ratio, alcohol user, smoking status, ideal physical activity, healthy eating index (HEI) score, daily calorie intake, breakfast skipping, and diet record days;

‡ Model 3: further adjusted (from Model 2) for duration of diabetes, diabetes medication use, self-reported hypertension, hypercholesterolemia, and CVD, and self-reported hypertension, hypercholesterolemia medication use;

§ Model 4: further adjusted (from Model 3) for HbA1c, HOMA2_IR, systolic blood pressure, diastolic blood pressure, total cholesterol,triglyceride, high density lipoprotein, low density lipoprotein, and eGFR.

# Supplementary Table 4. Stratified analyses of the associations (hazard ratios, 95% CIs) between eating frequency and all-cause mortality among participants with diabetes in NHANES 1999–2014

|  | Eating frequency  (per 1 time increment) | Eating frequency < 3 | Eating frequency = 3 | Eating frequency = 4 | Eating frequency > 4 | P trend | P interaction |
| --- | --- | --- | --- | --- | --- | --- | --- |
| Age (years) | | | | | | | 0.346 |
| ≤ 65 | 0.82 (0.69,0.97) | 1.00 | 0.77 (0.47,1.24) | 0.74 (0.47,1.19) | 0.47 (0.26,0.88) | 0.030 |  |
| >65 | 0.95 (0.84,1.07) | 1.00 | 1.01 (0.57,1.77) | 0.79 (0.48,1.31) | 0.98 (0.56,1.72) | 0.284 |  |
| Gender | | | | | | | 0.927 |
| Male | 0.87 (0.75,1.00) | 1.00 | 1.17 (0.70,1.96) | 0.95 (0.55,1.63) | 0.73 (0.41,1.27) | 0.049 |  |
| Female | 0.91 (0.75,1.10) | 1.00 | 0.54 (0.31,0.93) | 0.50 (0.29,0.85) | 0.56 (0.31,1.03) | 0.344 |  |
| Race/ethnicity | | | | | | | 0.989 |
| White | 0.89 (0.77,1.04) | 1.00 | 1.02 (0.61,1.7) | 0.89 (0.52,1.52) | 0.74 (0.41,1.35) | 0.119 |  |
| Non-White | 0.85 (0.73,1.00) | 1.00 | 0.69 (0.43,1.1) | 0.57 (0.35,0.92) | 0.55 (0.32,0.95) | 0.039 |  |
| Alcohol user | | | | | | | 0.161 |
| Yes | 0.81 (0.71,0.93) | 1.00 | 0.98 (0.64,1.51) | 0.66 (0.42,1.01) | 0.59 (0.36,0.99) | 0.001 |  |
| No | 0.94 (0.78,1.13) | 1.00 | 0.60 (0.34,1.04) | 0.70 (0.41,1.20) | 0.58 (0.31,1.11) | 0.622 |  |
| Ideal physical activity | | | | | | | 0.131 |
| Yes | 0.82 (0.68,0.99) | 1.00 | 0.98 (0.49,1.93) | 0.68 (0.35,1.32) | 0.64 (0.32,1.27) | 0.026 |  |
| No | 0.90 (0.81,1.00) | 1.00 | 0.87 (0.57,1.34) | 0.81 (0.53,1.23) | 0.69 (0.43,1.10) | 0.057 |  |
| Smoking status | | | | | | | 0.649 |
| Never smoker | 0.84 (0.71,1.00) | 1.00 | 0.61 (0.38,0.96) | 0.57 (0.35,0.94) | 0.42 (0.24,0.74) | 0.035 |  |
| Ever smoker | 0.92 (0.77,1.09) | 1.00 | 0.97 (0.55,1.71) | 0.86 (0.46,1.62) | 0.79 (0.39,1.63) | 0.326 |  |
| Current smoker | 0.78 (0.61,1.01) | 1.00 | 0.70 (0.33,1.47) | 0.40 (0.19,0.84) | 0.50 (0.25,1.03) | 0.061 |  |
| BMI, kg/m^2^ | | | | | | | 0.316 |
| ≤ 30 | 0.94 (0.84,1.06) | 1.00 | 1.09 (0.55,2.14) | 0.94 (0.51,1.74) | 0.91 (0.49,1.70) | 0.269 |  |
| > 30 | 0.82 (0.70,0.94) | 1.00 | 0.72 (0.47,1.11) | 0.62 (0.39,0.98) | 0.48 (0.28,0.83) | 0.007 |  |
| Diabetes duration (years) | | | | | | | 0.954 |
| ≤ 10 | 0.87 (0.75,1.01) | 1.00 | 0.81 (0.53,1.25) | 0.67 (0.42,1.07) | 0.61 (0.34,1.11) | 0.049 |  |
| > 10 | 0.86 (0.73,1.01) | 1.00 | 0.79 (0.45,1.39) | 0.69 (0.40,1.20) | 0.60 (0.33,1.10) | 0.075 |  |

Note:

All the model adjusted for age, sex, race/ethnicity, BMI, education level, family income-poverty ratio, alcohol user, smoking status, ideal physical activity, healthy eating index (HEI) score, daily calorie intake, breakfast skipping, diet record days, duration of diabetes, diabetes medication use, self-reported hypertension, hypercholesterolemia, and CVD, self-reported hypertension, hypercholesterolemia medication use, HbA1c, HOMA2_IR, systolic blood pressure, diastolic blood pressure, total cholesterol, triglyceride, high density lipoprotein, low density lipoprotein, and eGFR, with exception of stratifying factors.

# Supplementary Table 5. Stratified analyses of the associations (hazard ratios, 95% CIs) between eating frequency and CVDs-related mortality among participants with diabetes in NHANES 1999–2014

|  | Eating frequency  (per 1 time increment) | Eating frequency < 3 | Eating frequency = 3 | Eating frequency = 4 | Eating frequency > 4 | P trend | P interaction |
| --- | --- | --- | --- | --- | --- | --- | --- |
| Age (years) | | | | | | | 0.834 |
| ≤ 65 | 0.76 (0.52,1.11) | 1.00 | 0.77 (0.28,2.10) | 0.47 (0.19,1.14) | 0.54 (0.19,1.54) | 0.156 |  |
| >65 | 0.73 (0.56,0.95) | 1.00 | 0.92 (0.42,2.01) | 0.51 (0.22,1.18) | 0.46 (0.15,1.40) | 0.008 |  |
| Gender | | | | | | | 0.837 |
| Male | 0.76 (0.61,0.96) | 1.00 | 1.08 (0.55,2.12) | 0.55 (0.27,1.1) | 0.59 (0.27,1.27) | 0.010 |  |
| Female | 0.70 (0.45,1.09) | 1.00 | 0.17 (0.06,0.47) | 0.15 (0.05,0.4) | 0.14 (0.04,0.49) | 0.129 |  |
| Race/ethnicity | | | | | | | 0.319 |
| White | 0.87 (0.65,1.16) | 1.00 | 1.55 (0.68,3.53) | 1.02 (0.43,2.39) | 1.02 (0.34,3.06) | 0.313 |  |
| Non-White | 0.62 (0.45,0.86) | 1.00 | 0.46 (0.21,0.98) | 0.24 (0.12,0.49) | 0.24 (0.08,0.71) | 0.003 |  |
| Alcohol user | | | | | | | 0.420 |
| Yes | 0.74 (0.56,0.98) | 1.00 | 0.86 (0.43,1.71) | 0.41 (0.20,0.84) | 0.47 (0.18,1.22) | 0.022 |  |
| No | 0.66 (0.44,1.00) | 1.00 | 0.55 (0.17,1.80) | 0.42 (0.14,1.25) | 0.25 (0.06,0.97) | 0.056 |  |
| Ideal physical activity | | | | | | | 0.857 |
| Yes | 0.73 (0.54,0.99) | 1.00 | 1.06 (0.31,3.66) | 0.65 (0.19,2.16) | 0.46 (0.14,1.50) | 0.024 |  |
| No | 0.75 (0.58,0.97) | 1.00 | 1.02 (0.52,2.01) | 0.51 (0.25,1.05) | 0.59 (0.25,1.38) | 0.019 |  |
| Smoking status | | | | | | | 0.565 |
| Never smoker | 0.73 (0.53,0.99) | 1.00 | 0.34 (0.13,0.85) | 0.27 (0.11,0.63) | 0.27 (0.10,0.70) | 0.048 |  |
| Ever smoker | 0.76 (0.54,1.05) | 1.00 | 1.35 (0.60,3.04) | 0.70 (0.28,1.72) | 0.71 (0.19,2.61) | 0.099 |  |
| Current smoker | 0.59 (0.35,0.98) | 1.00 | 0.94 (0.14,6.07) | 0.26 (0.03,1.95) | 0.33 (0.06,1.88) | 0.023 |  |
| BMI, kg/m2 | | | | | | | 0.764 |
| ≤ 30 | 0.76 (0.59,0.96) | 1.00 | 0.78 (0.31,1.96) | 0.44 (0.18,1.10) | 0.48 (0.18,1.23) | 0.016 |  |
| >30 | 0.68 (0.49,0.95) | 1.00 | 0.75 (0.31,1.82) | 0.44 (0.19,1.00) | 0.40 (0.12,1.32) | 0.030 |  |
| Diabetes duration (years) | | | | | | | 0.981 |
| ≤ 10 | 0.75 (0.55,1.03) | 1.00 | 0.89 (0.43,1.84) | 0.42 (0.20,0.87) | 0.58 (0.19,1.78) | 0.056 |  |
| >10 | 0.78 (0.56,1.08) | 1.00 | 0.94 (0.33,2.66) | 0.72 (0.25,2.07) | 0.53 (0.16,1.71) | 0.157 |  |

Note:

All the model adjusted for age, sex, race/ethnicity, BMI, education level, family income-poverty ratio, alcohol user, smoking status, ideal physical activity, healthy eating index (HEI) score, daily calorie intake, breakfast skipping, diet record days, duration of diabetes, diabetes medication use, self-reported hypertension, hypercholesterolemia, and CVD, self-reported hypertension, hypercholesterolemia medication use, HbA1c, HOMA2_IR, systolic blood pressure, diastolic blood pressure, total cholesterol, triglyceride, high density lipoprotein, low density lipoprotein, and eGFR, with exception of stratifying factors.


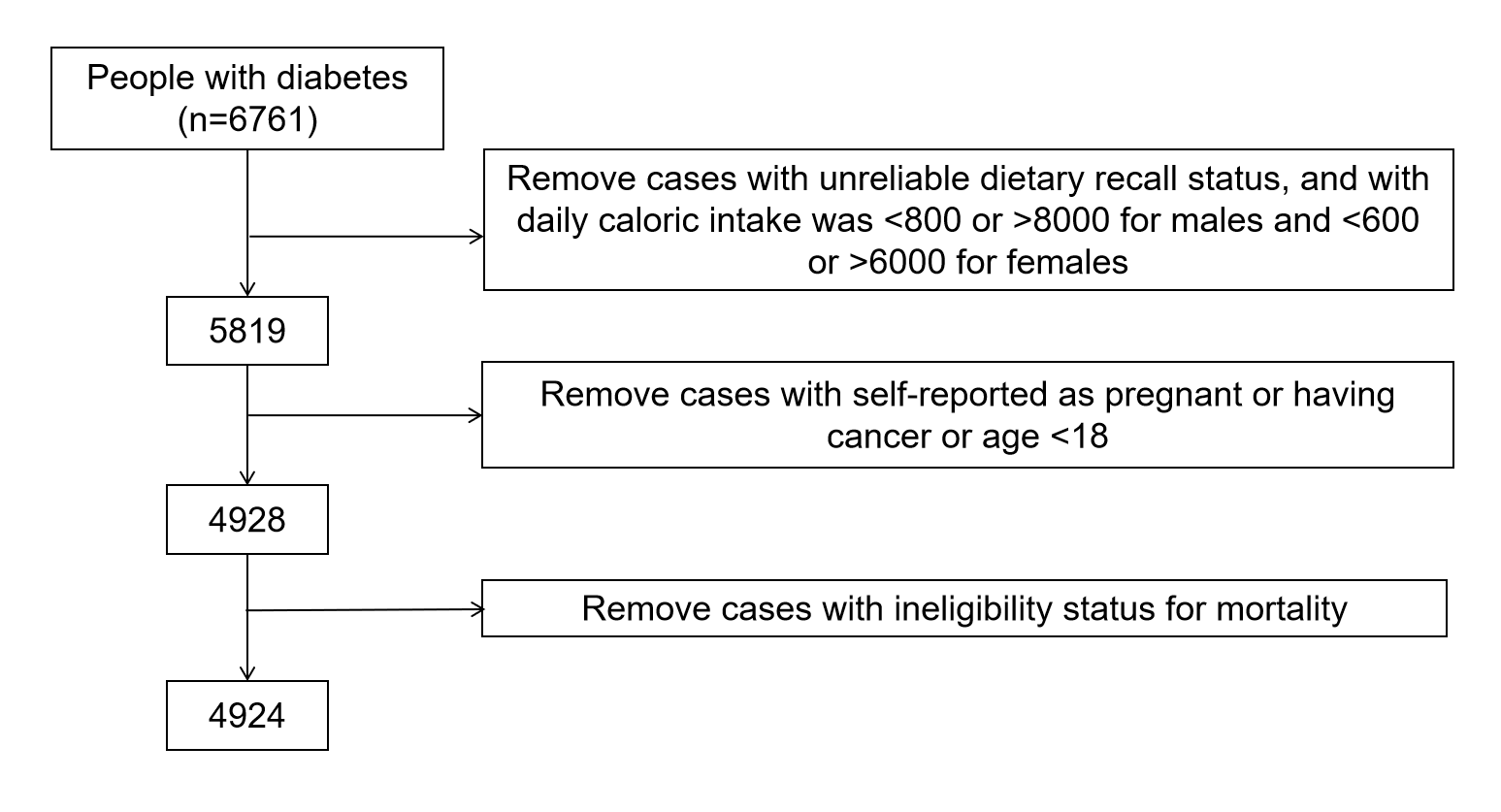


# Supplementary Figure 1. Algorithm for participant selection in the NHANES (1999-2014). Diabetes was defined as self-reported doctor diagnosis of diabetes, use of insulin or fasting glucose>= 7.0 mmol L-1 or glycated haemoglobin (HbA1c) level>= 6.5%, according to ADAs diabetes diagnostic criteria.
